# The chloroplast genome of a unicellular green alga strain isolated from the rubber processing wastewater

**DOI:** 10.1080/23802359.2020.1844090

**Published:** 2021-01-21

**Authors:** Bingying Han, Yaojia Mu, Deguan Tan, Shuai Ma, Lili Fu, Xuepiao Sun, Jiaming Zhang

**Affiliations:** a Institute of Tropical Bioscience and Biotechnology, MOA Key Laboratory of Tropical Crops Biology and Genetic Resources, Hainan Bioenergy Center, Chinese Academy of Tropical Agricultural Sciences, Haikou, China; b College of Life Sciences, Nanjing Agricultural University, Nanjing, China; c Hainan Institute for Tropical Agricultural Resources, Chinese Academy of Tropical Agricultural Sciences, Haikou, China

**Keywords:** Chlorella, complete chloroplast genome, wastewater, rubber

## Abstract

*Chlorella vulgaris* ITBBA3-12 has a role in the purification of the rubber processing wastewater. Its complete chloroplast genome contains 168369 bp, with a G + C content of 33.0%. A total of 147 genes were annotated, including 113 protein-coding genes, three rRNA (rrn23, rrn16, and rrn5) genes, and 31 tRNA genes. The significant feature of the chloroplast genome is that the genes encoding subunit V (petG), VI (petL), and apocytochrome f (petA) of the cytochrome b6/f complex are in triplicate, which was not observed in the other *C. vulgaris* strains. Phylogenetic analysis using the chloroplast genomes of Chlorophyta species indicated that ITBBA3-12 is closely related to *C. vulgaris* strain UTEX259 and NJ-7, and they clustered in the *Chlorella* lineage.

The *Chlorella vulgaris* strain ITBBA3-12 was isolated from a wastewater pond of rubber processing located in Danzhou city, Hainan Province, China with geospatial coordinates N19°30′59″, E109°29′43″. It is stored at the ClonBank of Institute of Tropical Bioscience and Biotechnology at −80 °C in 15% glycerol. Its mitochondrion genome has been reported previously (Hu et al. 2020). This strain is tolerate to relatively dark environment in the blackish water, therefore, its chloroplast genome is of great interest.

The genomic DNA was isolated as previously described (Ma et al. 2020; Yu et al. 2020), and sequenced with both PacBio RSII and Illumina Hiseq 2500 platforms at Genoseq (Wuhan, China). The chloroplast genome was assembled and corrected using CANU (Koren et al. 2017) and GATK (Zhu et al. 2015), respectively, and deposited in the Genome Warehouse in National Genomics Data Center, Chinese Academy of Sciences, under accession number GWHANRG00000000, and in GenBank under accession number MT920676.

The circular chloroplast genome has a length of 168,369 bp, bigger as compared to the chloroplast genomes of *C. vulgaris* strain C-27 (150,613 bp, MK948100) and strain NJ-7 (154,201 bp, NC_045362). The GC content is 33.0%, higher than the two sister strains (31.6%). A total of 147 genes were annotated, including 113 protein-coding genes, three rRNA (rrn23, rrn16, and rrn5) genes, and 31 tRNA genes. The protein-coding genes are mainly involved in photosynthesis (rbcL, psaA, psaB, psaC, psaI, psaJ, psbA, psbB, psbC, psbD, psbE, psbF, psbH, psbI, psbJ, psbL, psbN, psbT, psbZ, chlB, chlI, chlL, chlN, atpA, atpB, atpE, atpF, atpH, atpI, ycf3, ycf4, ycf62, 2 ycf78, and accD), protein synthesis (rpl12, rpl14, rpl16, rpl19, rpl2, rpl20, rpl23, rpl36, rpl5, rps11, rps12, rps14, rps18, rps19, rps2, rps3, rps4, rps7, rps8, rps9, and infA), RNA synthesis (rpoA, rpoB, rpoC1, and rpoC2), cytochrome related (3 petA, 3 petL, 3 petG, petB, petD, and ccsA), sulfate transport (cysA and cysT), hydrolyzes (I-CvuI and clpP), cell division (ftsH), and cell envelop (cemA). 30 hypothetical protein-coding genes were also identified. The 31 tRNA genes cover the transfer of all 20 amino acids, in which five are tRNA-Leu genes. The significant feature of the chloroplast genome is that the genes encoding the apocytochrome f (petA), subunit V (petG), and subunit VI (petL) of the cytochrome b6/f complex are in triplicate, which was not observed in the other *C. vulgaris* strains. The cytochrome b6/f complex functions in connecting photosystem II and photosystem I, and plays essential roles in regulating the electron transport (Boekema et al. 1994). The triplication of the cytochrome b6/f complex subunit genes may explain the wastewater tolerance in the strain ITBBA3-12.

Phylogenetic analysis using the chloroplast genomes of Chlorophyta species indicated that strain ITBBA3-12 is closely related to *C. vulgaris* strain UTEX259 and NJ-7, and they clustered in the *Chlorella* lineage with 100% bootstrap support (Figure 1).

**Figure 1.**
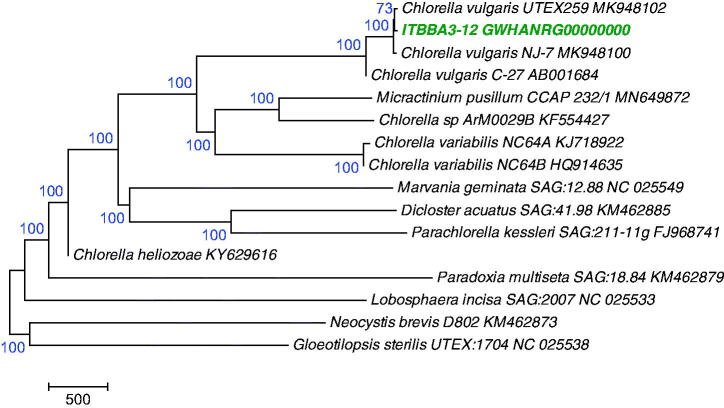
A Maximum Likelihood tree of Trebouxiophyceae species based on chloroplast genomes. The tree was conducted in MEGA7 (Kumar et al. 2016) and was rooted with a representative species from Chlorophyceae (KM462873) and a species from Ulvophyceae (NC_025538), and was bootstrap-tested.

## Data Availability

The whole genome sequence data reported in this paper has been deposited in the Genome Warehouse in National Genomics Data Center (Members 2019), Beijing Institute of Genomics (China National Center for Bioinformation), Chinese Academy of Sciences, under accession number GWHANRG00000000 that is publicly accessible at https://bigd.big.ac.cn/gwh. It is also available in GenBank (MT920676).
